# Ultrasound evaluation of cardiac and diaphragmatic function at different positions during a spontaneous breathing trial predicting extubation outcomes: a retrospective cohort study

**DOI:** 10.1186/s12880-024-01357-7

**Published:** 2024-08-15

**Authors:** Ling Luo, Yidan Li, Lifang Wang, Bing Sun, Zhaohui Tong

**Affiliations:** 1 Department of Respiratory and Critical Care Medicine, Beijing Jishuitan Hospital, Capital Medical University, Beijing, China; 2 Department of Ultrasound, Beijing Chaoyang Hospital, Capital Medical University, Beijing, China; 3 Epidemiology Research Center, Beijing Jishuitan Hospital, Capital Medical University, Beijing, China; 4 Department of Respiratory and Critical Care Medicine, Beijing Institute of Respiratory Medicine, Beijing Chaoyang Hospital, Capital Medical University, NO. 8 Gongren Tiyuchang Nanlu, Chaoyang District, Beijing, China

**Keywords:** Extubation, Echocardiography, Diaphragmatic ultrasound, Re-intubation, Cardiac dysfunction

## Abstract

**Background:**

The ratio (E/Ea) of mitral Doppler inflow velocity to annular tissue Doppler wave velocity by transthoracic echocardiography and diaphragmatic excursion (DE) by diaphragm ultrasound have been confirmed to predict extubation outcomes. However, few studies focused on the predicting value of E/Ea and DE at different positions during a spontaneous breathing trial (SBT), as well as the effects of △E/Ea and △DE (changes in E/Ea and DE during a SBT).

**Methods:**

This study was a reanalysis of the data of 60 difficult-to-wean patients in a previous study published in 2017. All eligible participants were organized into respiratory failure (RF) group and extubation success (ES) group within 48 h after extubation, or re-intubation (RI) group and non-intubation (NI) group within 1 week after extubation. The risk factors for respiratory failure and re-intubation including E/Ea and △E/Ea, DE and △DE at different positions were analyzed by multivariate logistic regression, respectively. The receiver operating characteristic (ROC) curves of E/Ea (septal, lateral, average) and DE (right, left, average) were compared with each other, respectively.

**Results:**

Of the 60 patients, 29 cases developed respiratory failure within 48 h, and 14 of those cases required re-intubation within 1 week. Multivariate logistic regression showed that E/Ea were all associated with respiratory failure, while only DE (right) and DE (average) after SBT were related to re-intubation. There were no statistic differences among the ROC curves of E/Ea at different positions, nor between the ROC curves of DE. No statistical differences were shown in △E/Ea between RF and ES groups, while △DE (average) was remarkably higher in NI group than that in RI group. However, multivariate logistic regression analysis showed that △DE (average) was not associated with re-intubation.

**Conclusions:**

E/Ea at different positions during a SBT could predict postextubation respiratory failure with no statistical differences among them. Likewise, only DE (right) and DE (average) after SBT might predict re-intubation with no statistical differences between each other.

**Supplementary Information:**

The online version contains supplementary material available at 10.1186/s12880-024-01357-7.

## Introduction

A spontaneous breathing trial (SBT) was a standard process by using to screen suitable patients for weaning from mechanical ventilation (MV). However, nearly 30% patients could succeed a SBT but fail extubation, who were identified as difficult-to-wean patients [1, 2]. It was always challenging to screen out patients at risk of extubation failure. Two main reasons for extubation failure were primary respiratory failure and cardiac dysfunction [3]. To avoid extubation failure, accurately assessing cardiac and diaphragmatic function during a SBT was essential, given their significant association with adverse outcomes.

Bedside ultrasound could be a convenient tool to assess cardiac function, diaphragmatic function and lung aeration in the weaning process [4]. Recently, a systematic review and meta-analysis including 11 studies reported that the ratio (E/Ea) of mitral Doppler inflow velocity (E) to annular tissue Doppler wave velocity (Ea) by transthoracic echocardiography (TTE) was significantly associated with weaning failure [5]. However, subgroup analysis revealed significant differences in these conclusions across different positions, with moderate heterogeneity. The guideline recommended that E/Ea could be assessed cardiac diastolic function at different positions, including septal, lateral and average [6]. However, there were few studies directly comparing with the effects of E/Ea at different positions during a SBT on extubation outcomes until now. Moreover, cardiac diastolic function might make a difference during a SBT in theory, indeed a SBT was a cardiovascular stress test in essence. Similarly, limited studies focused on the association between the changes in E/Ea during a SBT (△E/Ea) and extubation outcomes.

Currently, several systematic reviews and meta-analysis also showed diaphragm thickening fraction (DTF) and diaphragmatic excursion (DE) could help predict extubation failure, with significant heterogeneity [7–10]. Target patient, sample size, different positions (right, left and average), time points (before/after SBT or after extubation) might be the main reasons for heterogeneity. Some studies reported that DE at different positions were closely related to extubation outcomes [11–14]. However, there were few studied directly comparing with the effects of the diaphragm function at different positions during a SBT on extubation outcomes. Moreover, diaphragm potential reserve function was stimulated in case of spontaneous breath during a SBT. However, only some small studies reported that the changes of DE during a SBT (△DE) were associated with extubation outcomes [15, 16]. The clinical implications of △DE needed to be elaborated.

Clarifying the association of E/Ea and DE at different positions during a SBT with extubation outcomes had the potential to significantly save time and optimize efficiency for physicians and operators. In this study, we reanalyzed the data of 60 patients difficult weaning to reveal these associations. The original data were shown in Supplementary material 1. In the previous study, E/Ea (average) after SBT could help forecast respiratory failure within 48 h, and DE (average) after SBT could help anticipate re-intubation within 1 week in the respiratory failure group [17]. However, the primary goal in this study was to compare the predicting value of E/Ea (septal, lateral, average) among different groups during a SBT, as well as DE (right, left, average). The second aim was to manifest whether △E/Ea or △DE during a SBT was associated with extubation outcomes.

## Methods

### Ethical approval

This prospective cohort research was conducted at 4 intensive care units (ICUs) (RICU, SICU, CCU and EICU) in Beijing Chaoyang Hospital from November 2012 to February 2014. This research design was authorized by the institutional ethical review board of Beijing Chaoyang Hospital on Sep 24th, 2012 (No. 2012-ke-88). Prior to participation, informed agreement was secured from each patient’s immediate family.

### Patient selection

The attending physicians were responsible for assessing the intubated patients for readiness to wean. When the patients had been intubated for more than 48 h, a SBT was conducted. If the first SBT failed, they were defined as difficult-to-wean patients [1, 18]. Then, these patients could be identified as the eligible participants, only if they successfully passed a SBT in the end and were extubated during 24 h. Exclusion criteria included pregnant women, individuals under the age of 18 years, a history of diaphragm paralysis or cervical spine injury, pneumothorax or pneumomediastinum, prosthetic mitral valve or severe mitral stenosis, difficult ultrasonic windows, use of neuromuscular blocking agents within 48 h prior to enrollment, scheduled preventive noninvasive ventilation (NIV) following planned extubation or direct tracheostomy after SBT, and extubation failure due to upper airway obstruction.

### Study design

All participants completed a 30-minute SBT wearing a T-piece in a supine position at an incline of 30–45 degrees. Prior to the SBT, they were connected to a ventilator in a mode of pressure support ventilation [pressure support 10–12 cmH_2_O, positive end expiratory pressure 5 cmH_2_O, and proper fraction of inspired oxygen (FiO_2_) ≤ 40%]. Both TTE and diaphragm ultrasound were preformed sequentially before and after SBT by an experienced ultrasound specialist using a Vivid i ultrasound system (GE Medical Systems Israel Ltd) with a 3.0 MHz probe during 15 min. This research procedure was in line with the previous published study [17]. The criteria of readiness to wean and the failure of SBT were provided in Supplementary material 2.

All participants were monitored for the events of respiratory failure within 48 h, and in consequently they were divided into respiratory failure (RF) and extubation success (ES) groups. Similarly, all patients were divided into re-intubation (RI) or non-intubation (NI) groups depending on whether they required re-intubation within 1 week. Postextubation respiratory failure, NIV and the re-intubation should meet one of the following major criteria described in Supplementary material 2.

### Technical information

Diaphragmatic movement was assessed using ultrasound with a probe placed over the lower intercostal spaces. For the right in the right diaphragm, the probe was positioned in the right anterior axillary line to use the liver as an acoustic window. For the left diaphragm, the probe was positioned in the left midaxillary line using the spleen as an acoustic window. Two-dimensional mode was employed to optimize probe positioning and select appropriate scan lines for each hemidiaphragm. With the probe fixed on the chest wall, the ultrasound beam was directed at an angle of 70 degrees or more toward the diaphragmatic domes. M-mode ultrasound was then used to visualize the motion of structures along the selected line during respiration [11, 19, 20]. Unilateral diaphragmatic excursion (DE) was calculated as half of this summed vertical distance during inspiration and expiration, which was measured from baseline to vertex for each breath cycle. DE (average) was calculated as the average of DE (right) and DE (left) measurements. At least six measurements of DE were taken for each side and averaged. If breaths were irregular, at least ten measurements per side were required before averaging.

The left ventricular ejection fraction (LVEF) was evaluated using the biplane Simpson’s method from the apical two-chamber and four-chamber views [21]. Pulsed-wave Doppler echocardiography was used to analyze mitral valve inflow. This. allowed for the measurement of the early peak diastolic velocity (E) at the tip of the mitral valve leaflets. Pulse-wave tissue Doppler echocardiography was used to analyze the myocardial velocities, which were recorded at the septal and lateral sides of the mitral valve ring in the four-chamber view, respectively. The early diastolic velocities (Ea) were then measured from these recordings. Accordingly, E/Ea (septal) and E/Ea (lateral) were calculated. Ea (average) equaled half of the sum of the values of Ea (septal) and Ea (lateral), so E/ Ea (average) could be also calculated [22]. Five measurements were enough for regular sinus cardiac rhythm. In the case of atrial fibrillation, frequent atrial or ventricular premature beats, ventricular or supraventricular tachycardia, at least ten measurements were needed [23].

Patients’ characteristics were recorded, encompassing age, gender, body mass index, Acute Physiology and Chronic Health Evaluation (APACHE) II score upon admission to ICU, duration from onset to the first intubation and from the first intubation to extubation, presence of comorbidities, tidal volume (V_T_), rapid shallow breathing index (RR/V_T_), white blood cell (WBC), hemoglobin levels, albumin levels, plasma creatinine levels and Glasgow coma scale (GCS) on the day of extubation.

During a SBT, systolic blood pressure (SBP), diastolic blood pressure (DBP), heart rate (HR), percutaneous oxygen saturation (SpO2), respiratory rate (RR), mental status, diaphoresis, and the change of electrocardiograph (ECG) were recorded. Mean arterial pressure (MAP) was calculated according to the equation below. The data of TTE, diaphragm ultrasound, arterial blood gas analysis, lactic acid and NT-pro-BNP were also recorded. The changes of E/Ea and DE during a SBT were named as △E/Ea and △DE, respectively.

$$\Delta \mathrm{E} / \mathrm{Ea}=\mathrm{E} / \mathrm{Ea} \text { after } \mathrm{SBT}-\mathrm{E} / \mathrm{Ea} \text { before SBT }$$

$$\triangle \mathrm{DE}=\mathrm{DE} \text { after } \mathrm{SBT}-\mathrm{DE} \text { before } \mathrm{SBT}$$

$$\mathrm{MAP}=\mathrm{DBP}+1 / 3(\mathrm{SBP}-\mathrm{DBP})$$

### Statistical analysis

Statistical evaluations were carried out utilizing SPSS 17.0 (Inc., Chicago, IL, USA). The information was presented as either means ± standard deviation or as the median quartile [first–third]. To compare quantitative variables, we employed the Unpaired Student’s, Mann-Whitney, and the paired Wilcoxon tests. Differences in measured data were evaluated using one-way analysis of variance. The Chi-square test or the Fisher’s exact test was used for comparing proportions. Risk factors predicting respiratory failure within 48 h or re-intubation within 1 week were identified using the backward-Wald method and examined using a multivariate logistic regression model. The risk factors for respiratory failure and re-intubation including E/Ea and △E/Ea, DE and △DE at different positions were described in details, respectively. The area under curve (AUC) was determined using the receiver operating characteristic (ROC) curve. Statistical significance was set at *P* ≤ 0.05. The ROC curves of E/Ea (right, left, average) or DE (right, left, average) in different groups were compared with each other using MedCalc 15 software, respectively.

## Results

### General information

**Fig. 1:**
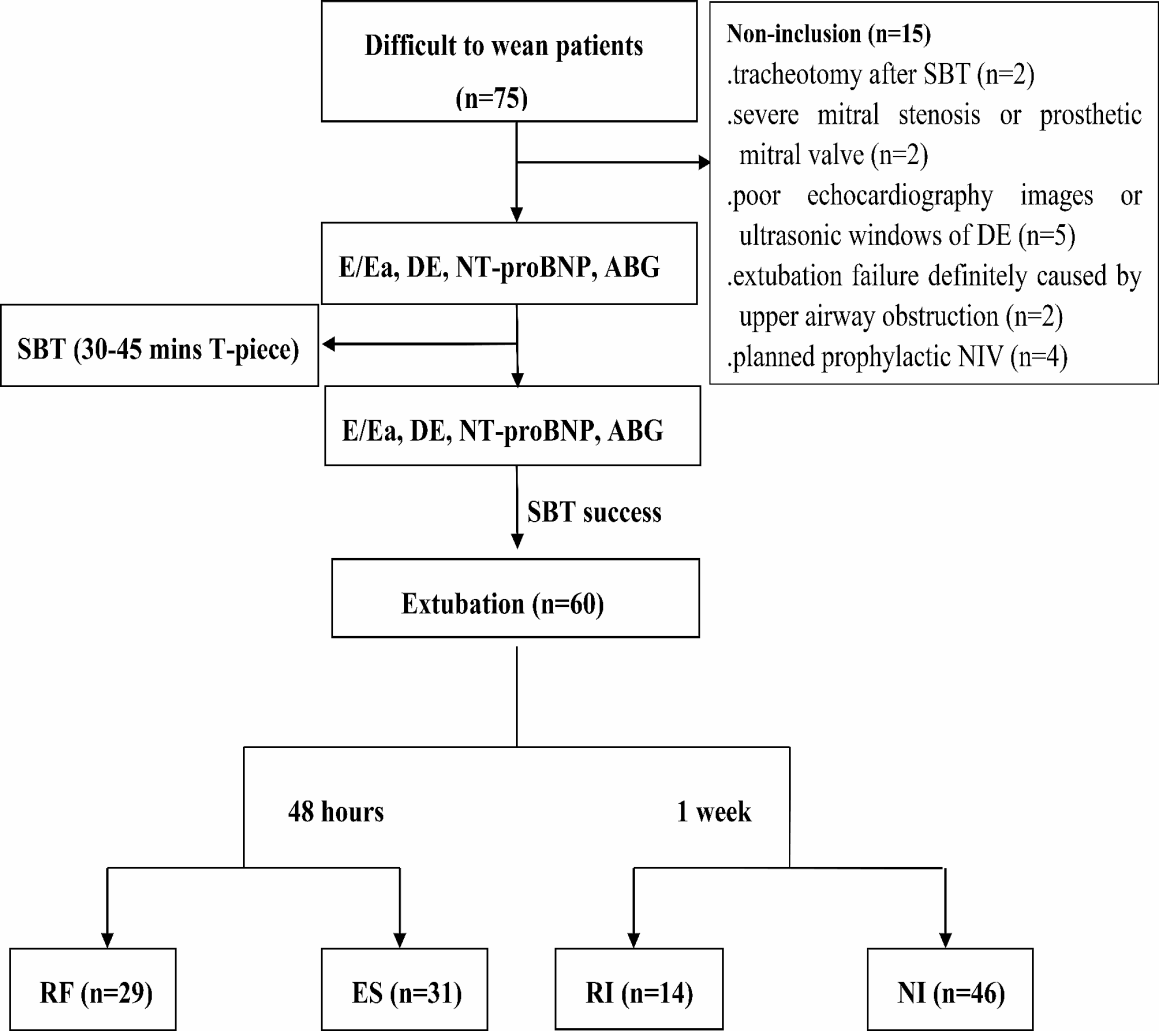
Flow chart. ABG, arterial blood gas analysis; DE, diaphragmatic excursion; E/Ea, the ratio of mitral Doppler inflow velocity (E) to annular tissue Doppler wave velocity (Ea); ES, extubation success; NI, non-intubation; NIV, noninvasive ventilation; NT-pro-BNP, N-terminal-pro-B-type natriuretic peptide; RF, respiratory failure; RI, re-intubation; SBT, spontaneous breathing trial

During the study period, a total of 60 patients were ultimately enrolled, and 15 cases were excluded for relevant reasons (Fig. 1). The participants included in the study underwent intubation for various reasons, including severe pneumonia (*n* = 19), chronic obstructive airway diseases (*n* = 10), cardiac events (*n* = 15), sepsis (*n* = 7), surgery (*n* = 8) and upper gastrointestinal hemorrhage (*n* = 1). Out of the total of 60 patients, 29 cases (48%) experienced postextubation respiratory failure within 48 h (respiratory failure group, RF group), while the remaining 31 cases (52%) were successfully extubated (extubation success group, ES group). Of 60 patients, 14 cases (23%) either required re-intubation or experienced mortality within 1 week (re-intubation group, RI group), and the remaining 46 cases were initiated on NIV or breathed spontaneously (non-intubation group, NI group) (Fig. 1).

### The changes of vital signs during a SBT

All patients succeeded to pass the SBT. SBP, MAP and RR after SBT were statistically higher than those before SBT (Table 1). There were no differences in DBP, HR and SPO_2_. 4 patients complained of slight sweating, and 3 patients felt mild fatigue or dyspnea. Atrial premature beat, ventricular premature beat, and mild ST segment descent occurred in one patient each during a SBT.

**Table 1:** The changes of vital signs during SBT

Vital signs	Before SBT	After SBT	*P* value
SBP	133 ± 20	139 ± 18	0.007
DBP	67 ± 12	68 ± 11	0.329
MAP	89 ± 12	92 ± 11	0.026
HR	88 ± 14	91 ± 18	0.100
SPO_2_	97.7 (96 ~ 99.8)	97.5 (96 ~ 100)	0.390
RR	22 ± 5	24 ± 5	< 0.001

Data are in median ± SD or median (IQR). DBP, diastolic blood pressure; HR, heart rate; MAP, Mean arteria pressure; RR, respiratory rate; SBT, spontaneous breathing trial; SPO_2_, percutaneous oxygen saturation

### Comparisons of E/Ea and △E/Ea in different groups during a SBT

**Fig. 2:**
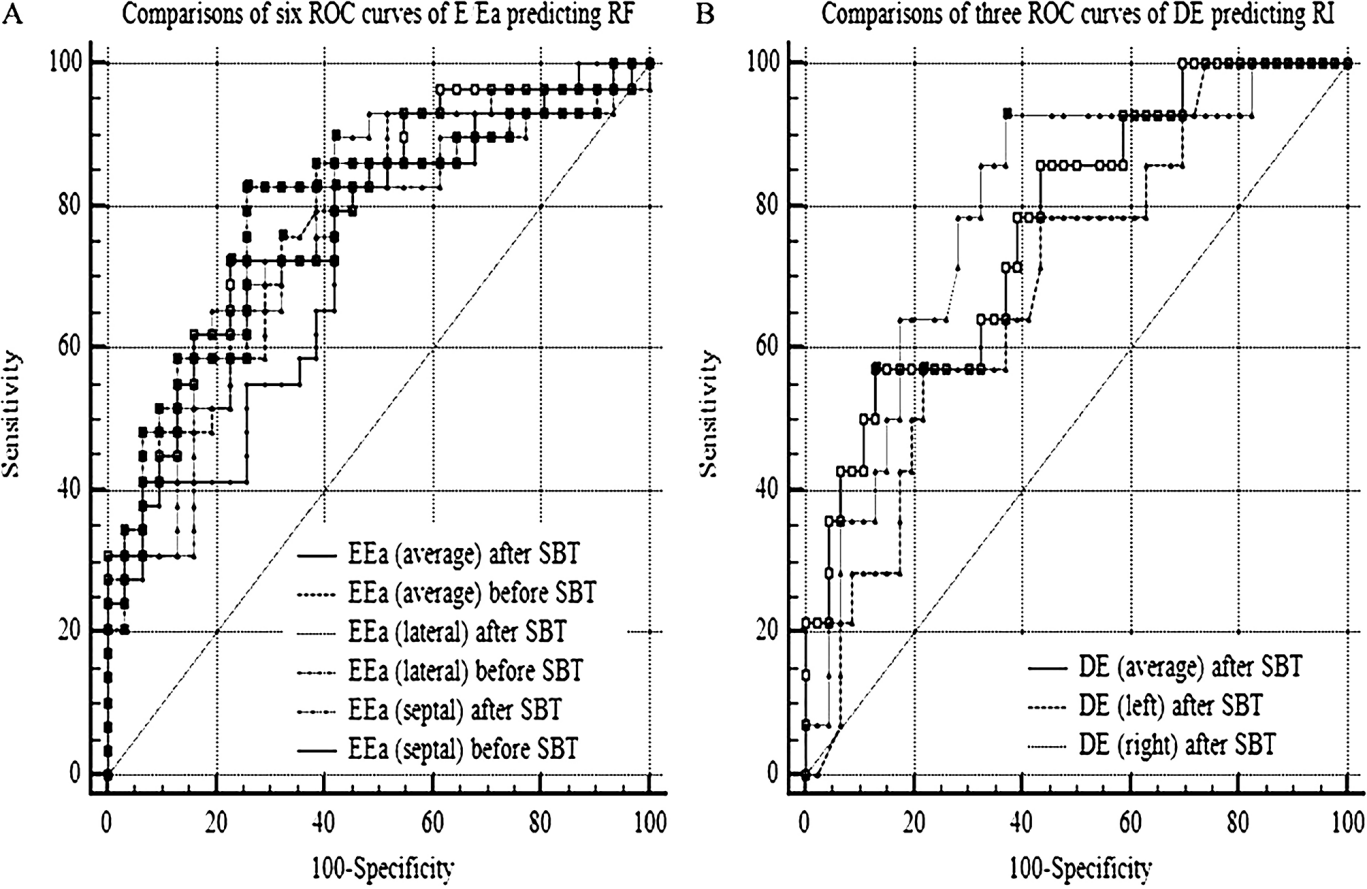
Comparisons of the ROC curves of E/Ea or DE at different positions during a SBT. **A**, Comparisons of the ROC curves of E/Ea during a SBT in six different groups showed no statistical differences among them (*p* > 0.05). **B**, No statistical differences were observed between the ROC curves of DE after SBT in three groups at different positions (*p* > 0.05). DE, diaphragmatic excursion; E/Ea, the ratio of mitral Doppler inflow velocity (E) to annular tissue Doppler wave velocity (Ea); RF, respiratory failure; RI, re-intubation; ROC, receiver operating characteristic; SBT, spontaneous breathing trial

Significant statistical differences in RF group and ES group were observed in the partial data of clinical characteristics, blood gas analysis, lactic acid levels, NT-pro-BNP levels, E/Ea and DE were thoroughly described in the previous study [17]. Multivariate logistic regression analysis revealed that E/Ea (septal, lateral, average) during a SBT were remarkably correlated with respiratory failure within 48 h. These risk factors including E/Ea in six groups were described in Supplementary material 3 (E1-E6). The parameters of the ROC curves of E/Ea were described in Table 2. Comparisons of ROC curves of E/Ea in six different groups showed no differences between each other (*P* > 0.05) (Fig. 2A). Further analysis exhibited that no differences in △E/Ea (septal, lateral, average) between RF and ES groups were found (Table 3).

**Table 2:** The parameters of ROC curves of E/Ea and DE at different positions during a SBT

Variables	95% CI	*P* value	Cut-off value	AUC	Sensitivity	Specificity
**Respiratory failure within 48 h**						
E/Ea (septal, before SBT)	0.589–0.828	< 0.001	> 12.4	0.720`	82.8%	58.1%
E/Ea (lateral, before SBT)	0.663–0.883	< 0.001	> 9.1	0.788	82.8%	74.2%
E/Ea (average, before SBT)	0.647–0.872	< 0.001	>10.6	0.774	75.9%	67.7%
E/Ea (septal, after SBT)	0.604–0.840	< 0.001	>13.4	0.734	82.8%	61.3%
E/Ea (lateral, after SBT)	0.657–0.878	< 0.001	>9.5	0.782	89.7%	58.1%
E/Ea (average, after SBT)	0.664–0.883	< 0.001	>12.5	0.789	72.4%	77.4%
**Re-intubation within 1w**						
DE (right, after SBT)	0.667–0.885	< 0.001	≤ 11.6	0.791	92.9%	63.0%
DE (left, after SBT)	0.560–0.805	0.013	≤ 10.3	0.693	57.14%	78.26%
DE (average, after SBT)	0.645–0.870	< 0.001	≤ 9.7	0.772	57.1%	87.0%

AUC, area under curve; CI, confidence intervals; DE, diaphragmatic excursion; E/Ea, the ratio of mitral Doppler inflow velocity to annulartissue Doppler wave velocity; ROC, receiver operating characteristic curve; SBT, spontaneous breathing trial

**Table 3:** The changes of E/Ea and DE during a SBT

	Respiratory failure within 48 h		Re-intubation within 1w	
	RF group	ES group	RI group	NI group
N (cases)	29	31	14	46
△E/Ea (septal)	0.4 ± 5.5	0.5 ± 2.0	0.4 ± 6.1	0.5 ± 3.3
△E/Ea (lateral)	1.5 ± 4.4	0.6 ± 1.9	1.2 ± 4.5	1.0 ± 3.0
△E/Ea (average)	1.0 ± 4.1	0.5 ± 1.8	0.9 ± 4.8	0.7 ± 2.4
△DE (right) (mm)	2.4 ± 4.6	0.9 ± 3.7	0.2 ± 2.9	2.1 ± 4.4
△DE (left) (mm)	2.2 ± 4.1	2.2 ± 4.4	0.6 ± 3.6	2.7 ± 4.3
△DE (average) (mm)	2.3 ± 3.5	1.6 ± 2.8	0.4 ± 2.8	2.4 ± 3.1*

Data are in median ± SD or median (IQR) or n (%). DE, diaphragmatic excursion; △DE, DE after SBT - DE before SBT; E, mitral Doppler early peak diastolic velocity; E/Ea, the ratio of mitral Doppler inflow velocity to annular tissue Doppler wave velocity; △E/Ea, E/Ea after SBT - E/Ea before SBT; NI, non-intubation; RI, re-intubation; SBT, spontaneous breathing trial. * *p* < 0.05 and † *p* < 0.001

### Comparisons of DE and △DE in different groups during a SBT

**Fig. 3:**
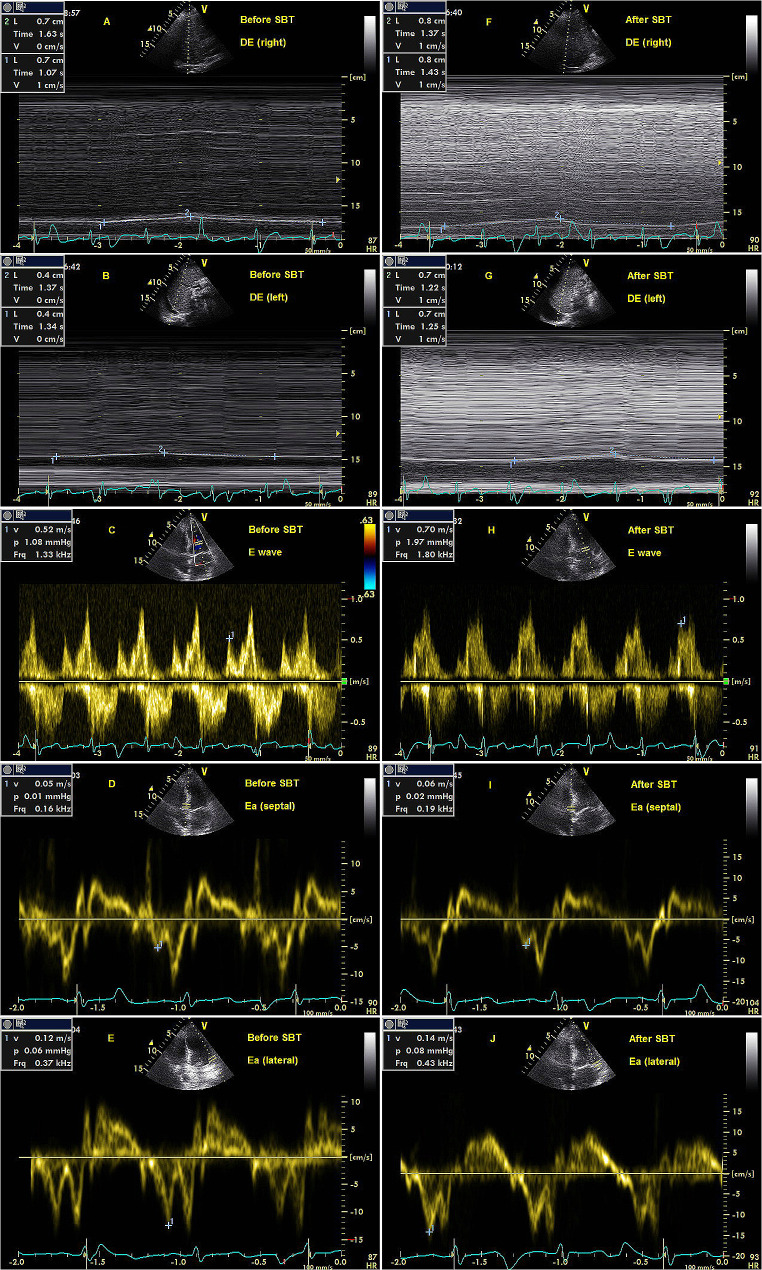
E/Ea and DE measurements during a SBT in a patient of re-intubation within 1 week. A, B, C, D, E represented the measurements before SBT, and F, G, H, I, J represented the measurements after SBT. (**A**) DE (right) was 7 mm. (**B**) DE (left) was 4 mm. (**C**) E was 52 cm/s. (**D**) Ea (septal) was 5 cm/s. (**E**) Ea (lateral) was 12 cm/s. (**F**) DE (right) was 8 mm. (**G**) DE (left) was 7 mm. (**H**) E was 70 cm/s. (**I**) Ea (septal) was 6 cm/s. (**J**) Ea (lateral) was 14 cm/s. Before SBT, the resultant DE (average) was 5.5 mm, and E/Ea (average) was 6.1. After SBT, the resultant DE (average) was 7.5 mm, and E/Ea (average) was 7. Respiratory failure did not occur within 48 h after extubation. However, the patient required re-intubation at the third day due to severe hypoxemia. DE: diaphragmatic excursion; E/Ea: the ratio of mitral Doppler inflow velocity (E) to annular tissue Doppler wave velocity (Ea); SBT: spontaneous breathing trial

Partial clinical characteristics were remarkably different in RI group and NI group (Table 4). Significant statistical differences were also observed in some data of laboratory markers and the indicators of diaphragm ultrasound (Table 5). Multivariate logistic regression analysis showed that only DE (right) and DE (average) after SBT were closely related to re-intubated within 1 week. These risk factors including DE (right or left or average) were described in Supplementary material 3 (E7-E9). The parameters of ROC curves of DE were described in Table 2. No differences were shown between DE after SBT in three groups at different positions (*P* > 0.05) (Fig. 2B). For instance, E/Ea during a SBT remained within the normal range in a patient diagnosed as severe acute pancreatitis, whereas DE during a SBT was observed to be low (Fig. 3). As a result, this patient was re-intubated at the third day after extubation due to severe hypoxemia.

**Table 4:** Clinical characteristics

	Re-intubation within 1w	
Group	RI group	NI group
N (cases)	14	46
**Demographic factors**		
Age (yrs)	70.1 ± 18.7	65.3 ± 20
Male	10 (71)	25 (54)
BMI (kg/m^2^)	22.3 ± 3.7	25 ± 4.9
**Comorbidities**		
Chronic respiratory diseases ^a^	8 (57)	18 (39)
Coronary heart disease	6 (43)	17 (37)
Myocardial infarction	3 (21)	14 (30)
Hypertension	8 (57)	28 (61)
Atrial fibrillation	4 (29)	7 (15)
Diabetes	4 (29)	14 (30)
Renal dysfunction	9 (64)	14 (30) *
Cerebral vascular diseases ^b^	5 (36)	5 (11)
**Clinical characteristics**		
APACHE II	25.6 ± 6	20.7 ± 5.5 *
Duration from onset to the first intubation (d)	6.7 ± 6.9	4.4 ± 3.7
Duration of the first intubation to extubation (d)	11.4 ± 8.9	7.9 ± 4.7
RR (breaths/min)	22.6 ± 5.7	21.2 ± 4
V_T_ (l)	0.39 ± 0.11	0.43 ± 0.1
RR/V_T_	63.9 ± 30.9	53.4 ± 18.2
GCS	12.7 (12 ~ 14.3)	13.9 (13 ~ 15)
WBC (×10^9^/l)	8.6 ± 3.8	11 ± 5
Hemoglobin (g/l)	85.4 ± 13.1	99.2 ± 20.9 *
Albumin (g/l)	27.8 ± 4.8	28.3 ± 3.9
Plasma creatinine (micromole/l)	141 (70 ~ 202)	111 (63 ~ 123)

Data are in median ± SD or median (IQR) or n (%). APACHE II, chronic health evaluation II; BMI, body mass index; GCS, Glasgow coma scale; NI, non-intubation; RI, re-intubation; RR, respiratory rate; RR/V_T_, rapid shallow breathing index; V_T_, tidal volume; WBC, white blood cell. ^a^ Asthma, obsolete tuberculosis, chronic obstructive pulmonary disease, bronchiectasis and interstitial lung disease. ^b^ Cerebral infarction, cerebral hemorrhage and subarachnoid hemorrhage. * *p* < 0.05, and † *p* < 0.001

**Table 5:** Laboratory and ultrasonic data

	Re-intubation within 1w	
	RI group	NI group
N (cases)	14	46
**Before SBT**		
PH	7.45 ± 0.05	7.46 ± 0.05
PaCO_2_ (mmHg)	47.3 ± 14.6	41.2 ± 8.4
PaO_2_/FiO_2_	309 ± 87	271 ± 78
Lactic acid (mmol/l)	1.2 (0.7 ~ 1.8)	1.1 (0.8 ~ 1.3)
NT-pro-BNP (pg/ml)	7232 (626 ~ 14,903)	3036 (262 ~ 2422)
LVEF (%)	58 ± 14	62 ± 12
E (cm/s)	92.7 ± 25.6	83.0 ± 22.2
E/Ea (septal)	16 ± 9.8	15.3 ± 5.6
E/Ea (lateral)	10.3 ± 5.9	10.8 ± 4.3
E/Ea (average)	12.3 ± 6.5	12.4 ± 4.6
DE (right) (mm)	8.6 ± 4.1	11.3 ± 4.7
DE (left) (mm)	9.8 ± 4.7	11.6 ± 6.1
DE (average) (mm)	9.2 ± 3.3	11.5 ± 4.0
**After SBT**		
PH	7.43 ± 0.05	7.46 ± 0.05
PaCO_2_ (mmHg)	48 ± 13.6	41.7 ± 8.4 *
PaO_2_/FiO_2_	314 ± 93	274 ± 89
Lactic acid (mmol/l)	1.2 ± 0.6	1.0 ± 0.4
NT-pro-BNP (pg/ml)	7307 (724 ~ 13,555)	2908 (266 ~ 2417)
E (cm/s)	94.7 ± 26.4	91.4 ± 21.3
E/Ea (septal)	16.3 ± 7.4	15.8 ± 5.7
E/Ea (lateral)	11.6 ± 7.6	11.9 ± 5.9
E/Ea (average)	13.2 ± 6.8	13.2 ± 5.4
DE (right) (mm)	8.7 ± 4	13.4 ± 5.3 *
DE (left) (mm)	10.4 ± 4.6	14.3 ± 5.9 *
DE (average) (mm)	9.6 ± 3.2	13.9 ± 4.5 *

Data are in median ± SD or median (IQR) or n (%). DE, diaphragmatic excursion; E, mitral Doppler early peak diastolic velocity; E/Ea, the ratio of mitral Doppler inflow velocity to annular tissue Doppler wave velocity; LVEF, left ventricular ejection fraction; NI, non-intubation; NT-pro-BNP, N-terminal-pro-B-type natriuretic peptide; PaCO_2_, arterial carbon dioxide tension; PaO_2_/FiO_2_, the ratio of arterial oxygen tension to fraction of inspired oxygen; RI, re-intubation; SBT, spontaneous breathing trial. * *p* < 0.05 and † *p* < 0.001

Further analysis revealed that only △DE (average) was remarkably higher in NI group than that in RI group (Table 3). However, multivariate logistic regression analysis showed that △DE was not associated with respiratory failure or re-intubation (Supplementary material 3 E10).

### Discussion

In this study, E/Ea (septal, lateral, average) during a SBT could predict respiratory failure within 48 h with no differences among them. △E/Ea at different positions was not associated with respiratory failure. Only DE (right) and DE (average) after SBT might predict re-intubation within a week with no statistical differences between each other. △DE at different positions was not associated with re-intubation.

### The changes of vital signs during a SBT

In clinical practice, identifying the cases of weaning failure or extubation failure was usually challenging. A SBT offered an effective method for identifying patients suitable for liberation from MV. However, its role was overestimated in difficult-to-wean patients. In this study, SBP and MAP and RR remarkably increased during a SBT. It implied that cardiac afterload and the work of respiratory muscles increased during a SBT. Although all patients successfully passed the SBT in this study, 29 cases (48%) still developed postextubation respiratory failure, and 14 cases (23%) required re-intubated or experienced mortality after extubation. These results revealed that a SBT was not a sensitive way to screen out patients with postextubation respiratory failure or re-intubation, especially in difficult-to-wean patients. It was necessary to explore more suitable methods to accurately predict extubation outcomes.

### Comparisons of E/Ea and △E/Ea in different groups during a SBT

In this study, E/Ea (septal, lateral, average) during a SBT had the similar predicting value of respiratory failure within 48 h. In 2020, Sanfilippo F et al. [5] performed a meta-analysis to manifest the association of weaning failure from MV with TTE parameters. They found that weaning failure was significantly associated to a higher E/Ea ratio, not LVEF and the ratio of mitral Doppler early peak diastolic velocity to late peak diastolic velocity (E/A). This meta-analysis also reported that significant differences between E/Ea in three groups including septal, lateral and average, with moderate heterogeneity (*P* = 0.04, *I*^*2*^ = 68.6%). In 2011, Papanikolaou J et al. [24] reported that E/Ea before SBT at different positions was all closely related to weaning outcomes. Due to high heterogeneity observed, we refrained from confirming the conclusions regarding the consistent predictive value of E/Ea at different positions. Until now, there had been few researches directly comparing E/Ea at different positions and various time points. Simultaneously, we also examined the history of myocardial infarction (17 cases, 28%) and lower EF% < 50% (10 cases, 17%) (Supplementary material 1), which often implied regional wall motion abnormality and might result in statistical differences in theory between E/Ea (lateral) and E/Ea (septal) predicting respiratory failure. It was necessary to address this issue for clarity. However, no significant differences were observed among the data from six groups. This could be reasonably explained by the fact that E/Ea at different positions during a SBT could all reflect cardiac diastolic dysfunction. Although the cut-off values of E/Ea might vary, their predicting value for respiratory failure could be similar across these variations.

In this study, △E/Ea at different positions was only slightly higher in RF group than that in ES group with no statistical differences. Different studies reached differing conclusions regarding △E/Ea. In 2010, Caille V et al. [25] found that there were no differences between E/Ea (lateral) before and after SBT in three groups according to the level of LVEF (> 50%, 35–50%, < 35%). However, Bedet A et al. [26] in 2019 revealed that during a SBT, echocardiographic indicators of left atrial pressure (E, E/A, and E/Ea) exhibited a significant increase in the failure group, whereas no such increase was observed in the success group. Similar results were reported in the studies conducted by Moschietto et al. [23] and E. Gerbaud et al. [27]. In essence, △E/Ea might reflect myocardial reserve to cope with the stress of SBT and be related to weaning-induced pulmonary edema, frequently accompanied with positive fluid balance and SBT-induced acute hypertension [28]. In this study, most patients routinely received the strategy of restricted liquid balance by giving furosemide and also kept stable blood pressure levels by continuous intravenous administration of nitrates if needed, which could potentially explain △E/Ea between RF and ES groups with no statistical differences.

### Comparisons of DE and △DE in different groups during a SBT

In this study, DE (right) after SBT and DE (average) after SBT were closely related to re-intubation. Meanwhile, no statistical differences were observed among DE after SBT at different positions (*P* > 0.05). According to the previous standard of DD (diaphragmatic dysfunction) defined as DE < 10 mm or paradoxical movements [11], we observed unilateral DD in 24 cases (12 cases on the right side, 12 cases on the left side) and bilateral DD in 5 cases (Supplementary material 1). Our findings suggested that assessing the DE (right) might be sufficient regardless of DD on the left side. It might be reasonably explained that the right diaphragm played a central role in overall diaphragm function. Additionally, our results also revealed that DE after SBT, rather than DE before SBT, was associated with re-intubation. DE before SBT could not give expression to diaphragm contractility truthfully, while 30 min T-tube SBT induced potential diaphragm contraction observed in DE after SBT.

Currently, few studies directly compared the impacts of DE at different positions. Additionally, most studies focused on DE after SBT, rather than DE before SBT [7–10]. Recently, there were three meta-analyses to assessing the diagnostic accuracy of DE to predict weaning outcome. In 2023, Parada-Gereda HM et al. [10] preformed a meta-analysis of 13 studies to confirm the predicting value of DE for successful weaning, with 7 studies focusing on DE (average), 6 studies on DE (right) and 1 study on DE (left). Significant heterogeneity was also evidenced in the sensitivity and specificity for DE (*I*^2^ = 65.1%, *P* = 0.001; *I*^2^ = 67.8%, *P* = 0.001, respectively). Similar conclusions were observed in studies conducted by Le Neindre A et al. in 2021 [7] and Li C et al. in 2018 [8]. The variation in positions was one of significant contributors to the heterogeneity, requiring further clarification. Our findings exhibited that DE (right) after SBT and DE (average) after SBT were associated with re-intubation within 1 week. This approach could save clinicians significant time, as obtaining the images of left diaphragm was more challenging compared to right diaphragm.

Furthermore, our results revealed statistical differences in △DE (average) between RI and NI groups. However, multivariate logistic regression analysis showed that △DE was not associated with re-intubation. In 2018, Palkar A et al. [16] showed that a decrease in DE of < 16.4% measured serially between assist control mode and SBT could predict extubation success better than DE alone with a sensitivity of 84.9% and a specificity of 65%. In 2019, Xia Zhang et al. [15] reported that in patients with chronic obstructive pulmonary disease, a cut-off value of △DE_30 − 5_ (the variation between 30 and 5 min during a SBT) > 0.16 cm might predict successful extubation with a sensitivity of 84% and a specificity of 83.3%, respectively. Our findings were not completely consistent with the two studies mentioned above. △DE reflected potential diaphragm reserve function, easily influenced by MV. Prolonged intubation duration was associated with a negative impact on diaphragm function, defined as ventilator-induced diaphragmatic dysfunction. In this study, the duration of MV in RI and NI groups were 10.0 (5.0, 16.3) vs. 7.0 (5.0, 9.3) days respectively (Supplementary material 1), notably longer than the duration reported in the two studies conducted by Palkar A et al. (3.5 ± 5.4 vs. 4.0 ± 3.2 days) and Xia Zhang et al. [3.7 (2.6 ~ 5.1) vs. 3.2 (1.9 ~ 4.5) days]. This could potentially be explained by the lack of significant association between △DE and re-intubation in this study.

Our results highlighted that the predicting value of E/Ea at different positions during a SBT for respiratory failure was consistent, as well as the similarity between DE (right) and DE (average) after SBT. These findings could streamline clinical decision-making and reduce the burden on physicians and operators.

Our study had several limitations. Firstly, we did not compare the impacts of DE and E/Ea in different modes and durations of SBT on extubation outcomes. The latest American Association for Respiratory Care (AARC) guideline indicated no significant differences between SBT using pressure support ventilation or SBT using a T-piece [29]. The previous American College of Chest Physicians (ACCP) guideline revealed no remarkably statistical differences in the weaning success rate between 30-min SBT and 120-min SBT [1]. However, there was still no consensus regarding the optimal modes and durations of SBT in assessing DE and E/Ea, as few studies directly compared these parameters under different conditions. Some studies suggested that DE should be conducted in the absence of ventilatory support [8, 30]. Therefore, future studies should be designed to clarity these questions. Secondly, DTF had been demonstrated as a superior indicator for assessing the active contractility of diaphragm, surpassing DE [8, 9]. It was also valuable to manifest the effects of DTE at different positions during a SBT on extuabtion outcomes in future study.

### Conclusions

E/Ea at different positions during a SBT could predict postextubation respiratory failure with no statistical differences among them. Only DE (right) and DE (average) after SBT might predict re-intubation with no statistical differences between each other.

### Electronic supplementary material

Below is the link to the electronic supplementary material.

Supplementary Material 1

Supplementary Material 2

Supplementary Material 3

## Data Availability

All data generated or analyzed during this study were included in this published article and its supplementary materials.

## Abbreviations

AARC: The American Association for Respiratory Care
ACCP: The American College of Chest Physicians
APACHE: Acute Physiology and Chronic Health Evaluation
AUC: Area Under Curve
V_T_: Tidal Volume
BNP: B-type Natriuretic Peptide
DBP: Diastolic Blood Pressure
DD: Diaphragmatic Dysfunction
DE: Diaphragmatic Excursion
△DE: The Changes of DE During a Spontaneous Breathing Trial
DTF: Diaphragm Thickening Fraction
E: Mitral Doppler Inflow Velocity
EA: Annular Tissue Doppler Wave Velocity
E/A: The Ratio of Mitral DOPPLER Early Peak Diastolic Velocity to Late Peak Diastolic Velocity
ECG: Electrocardiograph
E/EA: The Ratio of Mitral Doppler Inflow Velocity (E) to Annular Tissue Doppler Wave Velocity (EA)
△E/EA: The changes of E/EA During a Spontaneous Breathing Trial
ES Group: Extubation Success Group
FiO_2_: Proper Fraction of Inspired Oxygen
GCS: Glasgow Coma Scale
HR: Heart Rate
LVEF: Left Ventricular Ejection Fraction
ICU: Intensive Care Units
MAP: Mean Arterial Pressure
MV: Mechanical Ventilation
NI Group: Non-Intubation Group
NIV: Noninvasive Ventilation
NT-pro-BNP: N-Terminal-Pro-BNP
Or: Odds Ratio
RF Group: Respiratory Failure Group
RI Group: Re-Intubation Group
Roc: Receiver Operating Characteristic
RR: Respiratory Rate
RR/V_T_: Rapid Shallow Breathing Index
SBP: Systolic Blood Pressure
SBT: Spontaneous Breathing Trial
TTE: Transthoracic Echocardiography
V_T_: Tidal Volume
WBC: White Blood Cell
