# Supplementary material for: Ultrasound evaluation of cardiac and diaphragmatic function at different positions during a spontaneous breathing trial predicting extubation outcomes: a retrospective cohort study
Source: BMC Med Imaging. 2024 Aug 15;24:217. doi: 10.1186/s12880-024-01357-7 (PMC11328514; doi:10.1186/s12880-024-01357-7)
Supplement: Supplementary file 2 — Supplementary Material 2 [file 12880_2024_1357_MOESM2_ESM.docx]

**Ultrasound evaluation of cardiac and diaphragmatic function at different positions during a spontaneous breathing trial predicting extubation outcomes: a retrospective cohort study**

**Criteria**

All patients were connected to ventilator with pressure support ventilation before the spontaneous breathing trial (SBT). The criteria of readiness to wean: adequate cough, absence of excessive tracheobronchial secretion, resolution of disease acute phase for which the patient was intubated, stable cardiovascular status [heart rate (HR) ≤ 140 beats/min, systolic arterial pressure (SBP) 90 - 160 mmHg with no or minimal vasopressors], stable metabolic status, adequate oxygenation [percutaneous oxygen saturation (SpO_2_) > 90% on proper fraction of inspired oxygen (FiO_2_) ≤ 40% with positive end expiratory pressure (PEEP) ≤ 8 cmH_2_O], adequate mentation[[1](#_ENREF_1)].

A SBT was performed during a 30 minutes period using a T-piece while the patients lay in a supine position (30°-45°) with FiO_2_ ≤ 50%. SBT failure was defined at least one of the following reasons: agitation and anxiety, depressed mental status, cyanosis, obvious diaphoresis, severe arrhythmia including asystole or ventricular fibrillation or ventricular tachycardia or arrhythmia causing unstable hemodynamics, arterial pH < 7.32, arterial carbon dioxide tension (PaCO_2_) > 10 mmHg than baseline, arterial oxygen tension (PaO_2_) ≤ 60 mmHg at FiO_2_ ≥ 0.5, respiratory rate (RR) ≥ 35 breathes/min, HR > 140 beats/min or cardiac arrhythmia, SBP > 180 mmHg or < 90 mmHg[[1](#_ENREF_1), [2](#_ENREF_2)]. If a SBT was successful, the planned extubation was performed in the next 24 hours.

Postextubation respiratory failure was defined by at least two of the following criteria: arterial pH < 7.35 with PaCO_2_ > 45 mmHg or > 20% higher than baseline, RR >30 breaths/min or ≥ 50% higher than baseline, PaO_2_ < 60 mmHg or SpO_2_ < 90% at FiO_2_ ≥ 0.5, retraction of intercostal spaces, use of accessory respiratory muscles, or thoracic-abdominal paradoxical movement, decreased consciousness, agitation, or diaphoresis[[3-6](#_ENREF_3)]. Noninvasive ventilation (NIV) could be applied in the case of postextubation respiratory failure. The attending doctor decided whether to use NIV according to patients' conditions, consciousness and [tolerance](file:///C:\Users\lenovo\Desktop\chest\罗凌-chest\../../AppData/Local/Yodao/DeskDict/frame/20150728214337/javascript:void(0);) in the end.

The criteria of re-intubation were met one of the following major criteria (a): respiratory or cardiac arrest, persistent severe hypoxemia (the ratio of PaO_2_ to FiO_2_ ≤ 130 mmHg) in spite of NIV, hemodynamic instability with SBP ≤ 85mmHg despite adequate vasoactive drugs, severe cardiac arrhythmia; or (b) at least two of the following minor criteria: clinical signs of severe acute respiratory failure with RR persistently > 35 breaths/min, arterial pH ≤ 7.20, worsening of acute respiratory failure under NIV, clinical signs suggestive of severely decreased consciousness (eg, coma, stupor, delirium) under NIV, bronchial hypersecretion under NIV, development of other organ failure[[6](#_ENREF_6)]. The attending doctor decided whether or not to re-intubate in the end.

**References**

1. Boles JM, Bion J, Connors A, Herridge M, Marsh B, Melot C, Pearl R, Silverman H, Stanchina M, Vieillard-Baron A *et al*: **Weaning from mechanical ventilation**. *Eur Respir J* 2007, **29**(5):1033-1056.

2. MacIntyre NR, Cook DJ, Ely EW, Jr., Epstein SK, Fink JB, Heffner JE, Hess D, Hubmayer RD, Scheinhorn DJ, American College of Chest P *et al*: **Evidence-based guidelines for weaning and discontinuing ventilatory support: a collective task force facilitated by the American College of Chest Physicians; the American Association for Respiratory Care; and the American College of Critical Care Medicine**. *Chest* 2001, **120**(6 Suppl):375S-395S.

3. Keenan SP, Powers C, McCormack DG, Block G: **Noninvasive positive-pressure ventilation for postextubation respiratory distress: a randomized controlled trial**. *JAMA* 2002, **287**(24):3238-3244.

4. Esteban A, Frutos-Vivar F, Ferguson ND, Arabi Y, Apezteguia C, Gonzalez M, Epstein SK, Hill NS, Nava S, Soares MA *et al*: **Noninvasive positive-pressure ventilation for respiratory failure after extubation**. *N Engl J Med* 2004, **350**(24):2452-2460.

5. Ferrer M, Valencia M, Nicolas JM, Bernadich O, Badia JR, Torres A: **Early noninvasive ventilation averts extubation failure in patients at risk: a randomized trial**. *Am J Respir Crit Care Med* 2006, **173**(2):164-170.

6. Girault C, Bubenheim M, Abroug F, Diehl JL, Elatrous S, Beuret P, Richecoeur J, L'Her E, Hilbert G, Capellier G *et al*: **Noninvasive ventilation and weaning in patients with chronic hypercapnic respiratory failure: a randomized multicenter trial**. *Am J Respir Crit Care Med* 2011, **184**(6):672-679.
