# Supplementary material for: Ultrasound evaluation of cardiac and diaphragmatic function at different positions during a spontaneous breathing trial predicting extubation outcomes: a retrospective cohort study
Source: BMC Med Imaging. 2024 Aug 15;24:217. doi: 10.1186/s12880-024-01357-7 (PMC11328514; doi:10.1186/s12880-024-01357-7)
Supplement: Supplementary file 3 — Supplementary Material 3 [file 12880_2024_1357_MOESM3_ESM.docx]

**Ultrasound evaluation of cardiac and diaphragmatic function at different positions during a spontaneous breathing trial predicting extubation outcomes: a retrospective cohort study**

**Table E1 The variables including E/Ea (septal) before SBT predicting respiratory failure within 48h**

| Variables | OR | 95% CI | *P-*value |
| --- | --- | --- | --- |
| PaCO2 after SBT | 1.209 | 1.017-1.437 | 0.031 |
| E/Ea (septal) before SBT | 1.257 | 1.007-1.568 | 0.043 |

**Table E2 The variable including E/Ea (lateral) before SBT predicting respiratory failure within 48h**

| Variables | OR | 95% CI | *P-*value |
| --- | --- | --- | --- |
| E/Ea (lateral) after SBT | 1.476 | 1.062-2.050 | 0.020 |

**Table E3 The variables including E/Ea (average) before SBT predicting respiratory failure within 48h**

| Variables | OR | 95% CI | *P-*value |
| --- | --- | --- | --- |
| PaCO_2_ after SBT | 1.193 | 1.005-1.415 | 0.044 |
| E/Ea (average) before SBT | 1.504 | 1.053-2.147 | 0.025 |

**Table E4 The variables including E/Ea (septal) after SBT predicting respiratory failure within 48h**

| Variables | OR | 95% CI | *P-*value |
| --- | --- | --- | --- |
| PaCO_2_ after SBT | 1.201 | 1.018-1.417 | 0.030 |
| E/Ea (sepetal) after SBT | 1.258 | 1.032-1.534 | 0.023 |

**Table E5 The variable including E/Ea (lateral) after SBT predicting respiratory failure within 48h**

| Variables | OR | 95% CI | *P-*value |
| --- | --- | --- | --- |
| E/Ea (lateral) after SBT | 1.408 | 1.042-1.901 | 0.026 |

**Table E6 The variable including E/Ea (average) after SBT predicting respiratory failure within 48h**

| Variables | OR | 95% CI | *P-*value |
| --- | --- | --- | --- |
| E/Ea (average) after SBT | 1.415 | 1.045-1.916 | 0.025 |

**Table E7 The variables including DE (right) after SBT predicting re-intubation within 1w**

| Variables | OR | 95% CI | *P-*value |
| --- | --- | --- | --- |
| PaCO_2_ after SBT | 1.157 | 1.015-1.318 | 0.029 |
| DE (right) after SBT | 0.786 | 0.619-0.997 | 0.047 |

**Table E8 The variables including DE (left) after SBT predicting re-intubation within 1w**

| Variables | OR | 95% CI | *P-*value |
| --- | --- | --- | --- |
| PaCO_2_ after SBT | 1.130 | 1.012-1.262 | 0.030 |
| DE (left) after SBT | 0.890 | 0.740-1.070 | 0.216 |

**Table E9 The variables including DE (right) after SBT predicting re-intubation within 1w**

| Variables | OR | 95% CI | *P-*value |
| --- | --- | --- | --- |
| PaCO_2_ after SBT | 1.139 | 1.009-1.285 | 0.036 |
| DE (average) after SBT | 0.669 | 0.470-0.952 | 0.026 |

**Table E10 The variables including △DE (average) after predicting re-intubation within 1w**

| Variables | OR | 95% CI | *P-*value |
| --- | --- | --- | --- |
| APACHE II | 1.140 | 0.974-1.334 | 0.103 |
| Renal dysfunction | 3.814 | 0.594-24.475 | 0.158 |
| Hb | 0.946 | 0.897-0.997 | 0.037 |
| PaCO_2_ after SBT | 1.124 | 1.004-1.257 | 0.043 |
| △DE (average) | 0.441 | 0.638-1.217 | 0.441 |

APACHE II, chronic health evaluation II; DE, diaphragmatic excursion; E/Ea the ratio of mitral Doppler inflow velocity (E) to annular tissue Doppler wave velocity (Ea); OR, odds ratio; CI, confidence intervals; PaCO_2_, arterial carbon dioxide tension; SBT, spontaneous breathing trial.
